# Design and baseline characteristics of the PerfectFit study: a multicenter cluster-randomized trial of a lifestyle intervention in employees with increased cardiovascular risk

**DOI:** 10.1186/s12889-015-2059-9

**Published:** 2015-07-28

**Authors:** Tessa A. Kouwenhoven-Pasmooij, Bosiljka Djikanovic, Suzan J. W. Robroek, Pieter Helmhout, Alex Burdorf, M. G. Myriam Hunink

**Affiliations:** Department of Epidemiology, Erasmus Medical Center, Rotterdam, The Netherlands; Department of Public Health, Erasmus Medical Center, Rotterdam, The Netherlands; Department of Occupational Health, Erasmus Medical Center, Rotterdam, The Netherlands; Institute of Social medicine, Faculty of Medicine, University of Belgrade, Belgrade, Serbia; Centre – School of Public Health, Faculty of Medicine, University of Belgrade, Belgrade, Serbia; Staff Joint Health Care Division, Command Service Center, Ministry of Defense, Utrecht, The Netherlands; Department of Radiology, Erasmus Medical Center, Rotterdam, The Netherlands; Center for Health Decision Sciences, Harvard T.H. Chan School of Public Health, Boston, USA

**Keywords:** Motivational interviewing, Health risk assessment, Blended care, Work, Lifestyle change, Cardiovascular risk, General health, Cost-effectiveness, Decision aid

## Abstract

**Background:**

The prevalence of unhealthy lifestyles and preventable chronic diseases is high. They lead to disabilities and sickness absence, which might be reduced if health promotion measures were applied. Therefore, we developed the PerfectFit health promotion intervention with a “blended care”-approach, which consists of a web-based health risk assessment (HRA) including tailored and personalized advice, followed by motivational interviewing (MI). We hypothesize that adding MI to a web-based HRA leads to better health outcomes. The objective is to describe the design and baseline characteristics of the PerfectFit study, which is being conducted among employees with high cardiovascular risk in the military workforce, the police organization and an academic hospital.

**Methods:**

PerfectFit is a cluster randomized controlled trial, consisting of two arms. Based on cardiovascular risk profiling, done between 2012 and 2014, we included employees based on one or more risk factors and motivation to participate. One arm is the ‘limited’ health program (control) that consists of: (a) an HRA as a decision aid for lifestyle changes, including tailored and personalized advice, and pros and cons of the options, and (b) a newsletter every 3 months. The other arm is the ‘extensive’ program (intervention), which is additionally offered MI-sessions by trained occupational physicians, 4 face-to-face and 3 by telephone, and is offered more choices of health promotion activities in the HRA. During the follow-up period, participants choose the health promotion activities they personally prefer. After six and twelve months, outcomes will be assessed by online questionnaires. After twelve months the cardiovascular risk profiling will be repeated. The primary outcome is self-reported general health. Secondary outcomes are self-reported work ability, CVD-risk score, sickness absence, productivity loss at work, participation in health promotion activities, changes in lifestyle (smoking, alcohol consumption, physical activity, stress management) and body mass index. Furthermore, a process evaluation and an economic analysis will be performed.

**Discussion:**

Additional coaching using MI is expected to be a key factor for success of the web-based HRA in employees with increased cardiovascular risk. This “blended care”-approach may be an essential strategy for effective health promotion activities.

**Trial registration:**

Dutch Trial Register by registration number NTR4894, 14/11/2014.

## Introduction

### Background

Although life expectancy has significantly increased over the past decades in many countries worldwide, the prevalence of unhealthy lifestyles, preventable chronic diseases and disabilities is rising [1–3]. Combined with an ageing workforce, today’s sustainable employability and well-being of employees are under increasing pressure. Major risk factors for their well-being are adverse lifestyle habits, such as physical inactivity, unhealthy diet, smoking, alcohol and stress. These lifestyle behaviors are associated with chronic diseases, and among them, the most common are cardiovascular disease (CVD) and diabetes. These diseases not only produce high healthcare costs, but also high indirect costs to society due to productivity losses [4–6]. There is a clear need for health promotion interventions that are effective and feasible among employees.

Web-based health promotion interventions, such as a web-based health risk assessment (HRA), have been increasingly used [7–9]. They have the potential of a broad reach and, if appropriately designed and implemented, may promote health and well-being, reduce absenteeism, and increase work productivity [10–12], thus leading to a positive “return on investment” [13, 14]. A recent meta-analysis showed, however, that these intervention effects were found to decline after intervention completion. This report stated that there is a need for innovative techniques to help participants maintain their lifestyle changes [15]. A counselling technique that has shown promising results in changing and maintaining health behavior is Motivational Interviewing (MI) [16–23]. MI is a patient-centered coaching technique, and is based on four principles: showing empathy; addressing discrepancy between current behavior and an alternative lifestyle behavior; reinforcing the clients’ sense of self-efficacy; and respectfully dealing with the clients’ resistance to change [24]. Whereas an HRA informs a participant of their risks of CVD and the options for risk reduction, MI is used to help the participant evoke and strengthen intrinsic motivation for lifestyle changes by respecting individual preferences and autonomy. HRA complemented with MI could be considered a form of “blended care”. This type of care involves an internet approach and face-to-face care blended into one integrated treatment [25]. As of recently, the blended approach has been increasingly used to effectively influence changes in lifestyle behaviors [26] [27].

Based on a “blended care” approach, we developed and implemented an intervention study called PerfectFit. PerfectFit is aimed at employees who are 40 years or older, have high physical and mental work demands, and possess at least one risk factor for cardiovascular disease [28]. We hypothesized that adding MI to an HRA improves the effectiveness and sustainability in changing unhealthy lifestyles and reducing risk factors for chronic diseases [26]. The objective of this study was to find out whether adding MI to a web-based HRA leads to further improvement in the overall health status and in secondary outcomes such as participation in health activities, work ability, sickness absence and productivity at work, and lifestyle behavior. In this paper, a detailed description of the study design is presented, along with the baseline characteristics of participants. Short- and long-term results of the intervention, as well as a process and an economic evaluation will be presented in future papers.

## Methods

### Study design

The PerfectFit study is a cluster randomized control trial [29] (cRCT) among employees of three organizations, aimed to compare two groups: a purely internet-based approach (*limited or control* intervention) and a group that is exposed to the internet-based approach supplemented with MI (“the blended approach”, *extensive* intervention). The internet approach consists of a web-based HRA (later addressed as “HRA“) with tailored and personalized advice for health behavior change as well as pros and cons of the options for change. In the extensive intervention, the HRA is supplemented with face-to-face care provided by an (in-house) occupational physician (OP) using MI. The elements of the intervention are based on existing modules, previously evaluated RCTs [22, 30–32] and a recent meta-analysis [33]. Measurements were done at baseline and will be repeated after 6 and after 12 months.

Participants were included between 2012 and 2014 after providing written informed consent. There were no risks associated with participating in PerfectFit. Confidentiality was guaranteed during the study for all participants, as no information about the cardiovascular risk profiling, the HRA, or the coaching was provided to others than stated in the Participant Information Form. Ethical approval was obtained from the Medical Ethical Committee of Erasmus MC Rotterdam (METC) by registration number MEC 2012–459. The trial was registered on November 14^th^ of 2014 at the Netherlands Trial Register by registration number NTR4894.

After trial commencement, three OPs withdrew because of other priorities in work or due to personal issues, resulting in the loss of one military cluster and less inclusions than expected. In order to achieve sufficient power, we obtained approval of the METC to include an additional organization (i.e., an academic hospital).

### Study setting

The study is being performed among employees of three Dutch organizations: the military force, the police force, and an academic hospital. All organizations have an in-company occupational health center. Prior to the start of PerfectFit, all involved OPs of these health centers were introduced to the design and the goals of the PerfectFit study.

### Study population

The study population consisted of military personnel of ten Dutch military bases in different geographical regions in the Netherlands (*n* = 4207), executive personnel of three units of the police force in the western, central, and northern part of the Netherlands (*n* = 4086), and all health professionals of six wards (intensive cares and emergency rooms) of an academic hospital (*n* = 207). Based on previous data of 10,624 employees from a range of companies [34], we estimated 57.3 % (*n* = 5444) to be eligible, based on having at least one of three risk factors (obesity, smoking, lack of physical activity).

An entry point for recruitment of the study population was cardiovascular risk profiling (later in text: cardio screening). In the military force, all military employees of 40 years and over are expected to have this cardio screening once every three years. In the other two organizations, this type of screening is voluntary.

### Recruitment of participants

Aiming for optimal participation and commitment among participants and OPs, we obtained endorsement for this intervention study from both the boards of directors and the workers’ councils to perform this study. Recruitment in the military force started in 2012, in the police force in 2013, and in the hospital in 2014. Follow-up measurements will be completed by October 2015.

Information on the cardio screening and the PerfectFit study was repeatedly given in different formats (e.g., intranet publications, organizational magazine, and PerfectFit flyers, that were given out to applicants to the cardio screening). In the hospital, an additional information session was organized for the managers of the participating wards. Employees who were 40 years or older received the PerfectFit-flyer and a letter of invitation for the cardio screening signed by the highest manager. Participation in the PerfectFit study was voluntary and free of charge. The cardio screening and coaching sessions could be done during working hours, in contrast to completing the HRA and undertaking any individually chosen health activities.

### Inclusion criteria for the study

Participants in this study were 40 years and older who presented with at least one CVD risk factor during the baseline cardio screening. The age group 40 years and older was chosen for two reasons. Firstly, cardiovascular risk scores such as the Framingham Risk Score [35] and the European SCORE function start at the age of 40. Secondly, cardio screening is mandatory for military personnel in this age group [36]. The screening was performed by the OPs of the participating health centres.

The cardio screening consisted of three components: (1) a short questionnaire with questions related to personal lifestyle and family history; (2) anthropometric measurements; and (3) blood measurements. In a face-to-face session, the OP provided “usual care”-advice according to the applicable Dutch guidelines [37].

The short questionnaire used in the cardio screening consisted of eight questions on health behavior such as smoking (yes, no); meeting the Dutch physical activity norm of exercising five times a week at moderate intensity for at least half an hour (yes, no); family and personal history of cardiovascular diseases (yes, no), such as suffering from atrial fibrillation (yes, no); a first degree family member with angina pectoris or a history of heart attack (yes, no); and ever being diagnosed with diabetes (yes, no) or hypertension (yes, no). Participants were also asked whether they were being treated with prescription drugs for hypertension or heart problems (yes, no).

For blood pressure, weight, height and serum measurements, the OPs or their assistants used the instruments that are available for their daily practice. These instruments could vary between OPs, but were the same throughout the study. Systolic and diastolic blood pressures (in mmHg) were measured twice in a seated position at rest, and average blood pressure values were used. Waist circumference was measured halfway between the lower rib and the iliac crest, as is advised by the Dutch obesity recommendations for general practitioners [38]. Serum was analysed for total cholesterol, HDL, LDL, triglycerides (mmol/l), and glucose (mmol/l). If the glucose level exceeded 11.1, then also HbA1c (%) was measured.

These three components of the cardio screening provided entry points for the study, as it was used to identify employees at high risk for CVD. Inclusion criteria were met if a person had at least one of the following risk factors for CVD:

1. Angina or myocardial infarction in first degree relatives;
2. Physical inactivity, i.e. not meeting the guideline of physical activity at moderate intensity less than 30 min a day for 5 days per week or comparable effort;
3. Smoking;
4. Self-reported diabetes mellitus or random glucose ≥ 11.1 mmol/l;
5. Obesity, defined as BMI ≥ 30 kg/m^2^ and / or waist circumference ≥ 102 cm for men or BMI ≥ 30 kg/m^2^ and/or ≥ 88 cm for women.
6. Hypertension (diastolic value > 90 mm Hg or a systolic value > 140 mmHg) or the use of antihypertensive drugs;
7. Dyslipidaemia (total cholesterol ≥ 5 mmol/l or LDL cholesterol ≥ 2.5 mmol/l or triglycerides: ≥ 1.7,mmol/l or HDL cholesterol: ≤ 1.0 mmol/l).

**Fig. 1:**
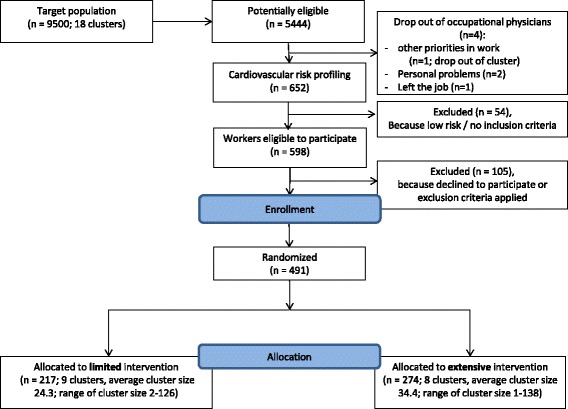
Flow of clusters and participants until allocation within the trial

Employees who met the above mentioned inclusion criteria were excluded from participation if they had (1) manifest CVD (history of myocardial infarction, heart failure, or cerebrovascular accident); (2) a terminal illness; or (3) a history of psychosis. A flowchart of participants is shown in Fig. 1.

### Study interventions

After the cardio screening at baseline, employees who were included in both the extensive and the limited study intervention groups, were invited by the OP to log on to the web-based HRA, by giving them a personal voucher-code.

#### Web-based HRA

**Fig. 2:**
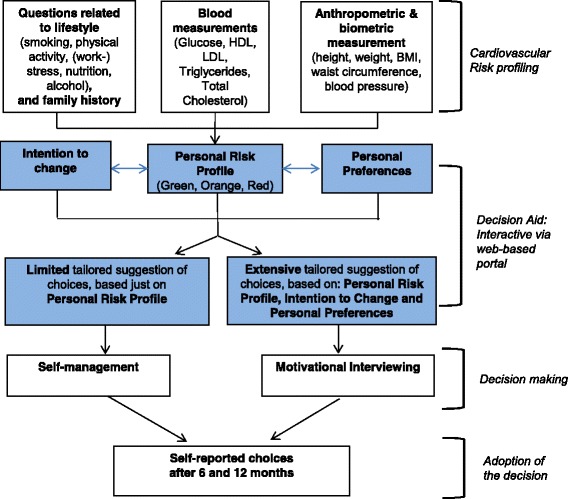
Flowchart of the interventions, including a web-based Health Risk Assessment (HRA) with tailored advice and suggestions of choices, and motivational interviewing

The web-based HRA consists of a web-based electronic questionnaire, including questions on lifestyle, work, family history, medical history, and motivation to change, which takes 30 to 45 min to fill out. Based on the answers on these questions, as well as on the baseline anthropometric and blood measurements that are integrated in the online system, the web-based HRA generates tailored and personalized advice to the participant, which are presented as low-risk (green), intermediate-risk (orange), or high-risk (red) profiles. Personalized advice includes a suggestion of choice out of health promotion activities, based on the participant’s risk profile, preferences and motivational aspects, according to the transtheoretical model of health behavior change [39]. These optional choices can be selected from a list of activities that are “usual care” for each organization. Prior to the inclusion period, this list was constructed by the OPs and the research team, and it includes health promotion activities on lifestyle items (i.e. sports facilities, dietician, psychologist). This web-based HRA can be considered as a patient decision aid, since it meets the six qualifying criteria [40]. The flowchart of the intervention is shown in Fig. 2.

For the participants in both intervention arms, follow-up questionnaires are conducted after 6 and 12 months, but without any tailored advice as feedback. In case of non-response to a questionnaire after two weeks, an automatic electronic reminder is sent to the employee. After another two weeks without response, the employee is contacted by a member of the research team reminding him/her to fill out the questionnaire and/or to provide assistance if needed.

### Limited (control) intervention

The limited health intervention program consists of the following elements:

- a web-based HRA (described above), including tailored and personalized advice for health behavior change, with a suggestion of choice out of three “usual care” health promotion activities for every identified risk factor. This is followed by usual care, according to Dutch OP guidelines [41].
- an electronic newsletter of approximately two pages, sent every three months. The newsletter includes general information on PerfectFit and on a healthy lifestyle.

### Extensive intervention

In the extensive intervention group, the limited intervention program is extended with personalized MI sessions with an OP, together with additional tailoring based on motivational elements in the web-based HRA and an additional motivational paragraph in the newsletters.

MI is conducted in the form of individual coaching sessions run by trained OPs. Altogether, they include seven motivational coaching sessions: three face-to-face sessions (30–45 min per contact) and four telephone contacts (15–30 min per contact) [42]. The MI training for OPs was organized as a continuous medical education (CME) session and after successful completion of the training OPs received credits. The training was free of charge and it consisted of three full days of group training by a certified MI trainer with 3 follow-up coaching sessions of 4 hours each [43]. During the training, OPs became familiar with the basic principles of MI and they practiced techniques needed for conducting MI. The aim was to elicit long-term healthy behavior in the participants.

To assure and maintain a high level of quality of MI during the intervention period, we conducted two quality assurance activities [44]. First, after every three months, each OP was asked to audio record the first face-to-face consultation with a participant, using a voice recorder. Recorded sessions were transcribed verbatim and analyzed using the validated Motivational Interviewing Treatment Integrity code (MITI) for the presence of core elements of the MI technique, such as reflexivity, open-questioning, and empathy [45, 46]. Scorings were done by the first author and a co-investigator in this study, who are both experienced MI-coaches and familiar with the scoring technique. Within a month after the recordings, the OPs received feedback on the quality of their MI-techniques. Second, after every MI session with participants, OPs had to fill out a form related to the MI session with a participant, such as if it was a face-to-face or telephone session, the amount of time it took, and what stage of change the participant was in (5 stages, ranging from pre-contemplation to maintenance).

The online advice within the HRA additionally includes tailoring based on motivational aspects such as intention to change and personal preferences, and it includes three more suggestions of choice for health promotion activities. In contrast to usual care, these additional suggestions of choice include e-health interventions, fitness centers with national coverage, and the use of an activity tracker (accelerometer). The accelerometer is offered for free and is primarily aimed at providing “biofeedback” to those participants who are physically inactive and who are motivated to improve their level of physical activity by using this small monitoring device during twelve consecutive weeks [47].

### Outcome measures

The following outcome measures will be taken into account:

- **Primary outcome measure:**

General health

At baseline, 6 months and 12 months, we measure (d) general health using the first question of the Short Form 36 Health Survey (SF-36) [48] (‘Overall, how would you rate your health?’), which has five possible answers, ranging from ‘poor’ to ‘excellent’.

- **Secondary outcome measures:**

Quality of life

Quality of life and its utility values will be calculated using the EuroQol 5 dimensions self-report questionnaire (EQ-5D) [49] and two domains from the SF-36 (physical functioning and vitality) [48]. The EQ-5D is a health status classification system consisting of five dimensions: mobility, self-care, usual activities, pain/discomfort, and anxiety/depression. We distinguish three levels for each dimension: no problems, moderate problems, and extreme problems. Within the SF-36, 4 questions are asked for physical functioning with 5 answering options, ranging from “totally agree” to “totally disagree”. For vitality 4 questions are asked with 6 answering options ranging from “all the time” to “never”.

#### CVD-risk score

CVD-risk will be estimated at baseline and after 12 months, using the European SCORE function (EuroSCORE), the Framingham Risk Score (FRS), and the QRISK2. The EuroSCORE [35, 50] estimates the 10-year risk for total fatal CVD-risk, based on age, sex, smoking, blood pressure, and total cholesterol. The FRS estimates the 10-year risk for CVD mortality and morbidity by adding hypertension treatment status, HDL-cholesterol, and diabetes status [36, 51, 52]. The QRISK2 [28] estimates the risk of CVD by adding ethnicity, diabetes, family history for angina or heart attack, chronic kidney disease, atrial fibrillation, blood pressure treatment, rheumatoid arthritis, cholesterol/HDL ratio, and body mass index.

#### Obesity

Obesity, measured with waist circumference (in cm) and Body Mass Index (BMI,kg/m2), was determined at baseline and will be repeated at 12 month follow-up.

#### Lifestyle

At baseline and after 6 and 12 months, current behavior is assessed for smoking, physical activity, alcohol consumption, and nutrition, measured as adherence to Dutch public health guidelines (yes/no), i.e., not smoking [53], being moderately physically active for 30 min on at least 5 days a week [54], not drinking more than 1 (women) or 2 (men) glasses of alcohol a day [55], eating at least 200 g of vegetables per day [55], and eating at least 2 pieces of fruit per day [55] .

At baseline, self-reported intentions to change behavior targeting smoking, physical activity, alcohol, dietary behavior or body weight will be assessed. For smoking, intentions to change are measured using dichotomous response scales (yes/no). For the other lifestyle items, the answering options are dichotomized into ‘I will start this month; I will start within 6 months; I will start, but I don’t know when’ and ‘I would like to, but I don’t have enough time; I would like to, but I can’t because of a disease, a disability or a doctor advised me not to; other’.

After 6 months, actual lifestyle behavior change will be measured by the number and type of health promotion activities they choose to participate in, within the 6 months after baseline (participants’ responsiveness to the intervention). Also the chosen mode of delivery (provided by employer or not) will be assessed.

#### Work ability

Work ability will be measured at baseline, 6 months and 12 months, using the Work Ability Index (WAI) questionnaire [56], which consists of seven dimensions: an individual’s (i) physical and (ii) mental demands related to work, (iii) diagnosed diseases, (iv) experienced work limitations due to disease, (v) sick leave in the previous 12 months, (vi) work ability prognosis, and (vii) mental resources. The WAI index is derived as the sum score of the ratings on each dimension. The range of the summative index is 7–49, which is categorized into “poor” (7–27), “moderate” (28–36), “good” (37–43), and “excellent” (44–49) work ability [57].

#### Productivity at work

Productivity at work and, if applicable, the reason for any self-reported loss in productivity was measured at baseline and will be repeated after 12 months. We use the Quantity and Quality (QQ) method [58], which is derived from the PRODISQ [59, 60]. On 10-point numerical scales, participants are asked how much work they performed during regular hours on their last regular workday and what the quantity of the work was compared to normal.

We also quantify to what degree employees are present at work but limited in their job performance due to any health problems, by using the short version of the Work Limitations Questionnaire (WLQ-8) [61–63]. The WLQ-8 consists of four dimensions: physical demands (2 items), time management (2 items), mental-interpersonal demands (2 items), and output demands (2 item). Individuals are asked to base their responses on their previous two weeks of work and to rate impairment on a 5-point scale ranging from “always” to “never” with an additional response item “does not apply to my job”.

### Process evaluation

After the intervention period, several process characteristics will be evaluated using the RE-AIM model [64, 65], which consists of the following five elements:

1. Reach (eligibility, number of cardio screenings, percentages of inclusions and exclusions, individuals’ characteristics, and compliance and determinants of the participants to the screening, HRA, MI-counselling sessions, and 6 and 12 months follow-up measurements);
2. Effectiveness (outcomes at 6 and 12 months);
3. Adoption (characteristics of OPs and clusters);
4. Implementation (amount, duration, timing and quality of MI-counselling sessions, satisfaction of participants with the sessions ); and
5. Maintenance (long-term implementation).

Participants’ opinions of the perceived usefulness of the web-based HRA, of the coaching, and of the impact on their lifestyle change will be evaluated at six months, on a 5-points Likert scale (from 1 = strongly disagree to 5 = strongly disagree). Participants’ satisfaction with the OP will also be measured at six months, using standard questions based on the PSOHQ [66] .

### Sample size and power calculation

The primary outcome measure in this study is general health. Based on a study of more than 4000 asymptomatic Dutch individuals [67], we assumed that the average value for general health score measured by the SF-36 within our target study population is 71 (SD 19, scale 0–100). Based on an intervention study that assessed general health, albeit with a less intensive intervention [68], we assume a relevant difference in the general health score between the intervention and control groups after 12 months of 10 % in favor of the intervention group. With a power of 80 %, a significance level of 5 %, two-tailed testing, a compensation for cluster randomization with an estimated intra-cluster correlation coefficient of 0.05, the assumption of similar groups, and with the intention of demonstrating superiority, approximately 220 participants per group are needed to demonstrate a difference in treatment effect of 10 % between the two groups. With an expected participation of 50 % (based on previous studies of health interventions for military personnel) and a loss to follow-up of 30 %, the RCT required inviting 2 × 634 individuals, so 1268 in total.

### Randomization, blinding, and allocation to interventions

Military bases, regional police forces, and hospital wards were randomly assigned as ‘clusters’ to one of the two intervention groups. A total of 18 clusters were randomized, which were equally divided in 9 clusters that were allocated to the limited intervention and 9 clusters that were allocated to the extensive intervention. In each of these clusters, one or more OPs were assigned to contribute to PerfectFit, based on shown interest. We chose for a cluster design to ensure that the OPs were only active within a single study arm. The organizational unit was the preferred cluster for two reasons. First, OPs from the same organizational unit were all trained to execute the intervention so that discussions among colleagues would not bias the results. Second, grouping participants from the same organizational unit into the same intervention group will prevent so-called “contamination effects” among participants, i.e., talking and sharing experiences about involvement in two different types of interventions, which might influence their behaviors [69, 70].

In the military force we constructed ten clusters consisting of military ground force army bases located in different regions in The Netherlands. Since the police force was facing a large reorganization with possible replacements of OPs from one organizational unit to another, only two clusters could be constructed, based on organizational units in different regions. Randomization in the academic hospital was performed at the ward level, creating a total of 6 clusters.

For all organizations, the OPs only perform work for the health center within that particular organizational unit. Clusters were ordered according to their sizes, i.e. the number of potential eligible participants. For each pair of clusters of similar size within one organization, one of the clusters was randomly allocated to the extensive intervention and the other to the limited intervention. Randomization took place after an OP from the unit had confirmed participation, and prior to the inclusion of individual participants. A researcher who was not otherwise involved in the trial used version 3.0.1 of The R Foundation for Statistical Computing for the randomization. Researchers, participating OPs, and participants were not blinded for the group allocation, since this was impossible given the nature of the intervention and the cluster design.

### Statistical analyses

Demographic and socio-economic characteristics of participants such as age, gender, education, profession, and income level, were collected at the beginning of the study. For the baseline characteristics of Tables 1 and 2, we used descriptive statistics to generate number and percentages for dichotomous and categorical variables, and to generate means and standard deviations of continuous variables. To get insight into the differences between groups, Chi-Square tests and ANOVA-tests were performed.

**Table 1:** Baseline characteristics of participants included in the PerfectFit-study according to organization ( *n* = 491)

	Organization 1	Organization 2	Organization 3	P value
	(Police)	(Military)	(Hospital)	
	*n* = 262 (53 %)	*n* = 170 (35 %)	*n* = 59 (12 %)	
Age, mean (sd)	52 (5.8)	49 (4.6)	53 (6.5)	<0.000^*^
Gender:				
Male	201 (82.7 %)	163 (98.2 %)	16 (27.6 %)	<0.000^*^
Educational level				
Low	39 (16.7 %)	16 (9.9 %)	11 (20 %)	<0.000^*^
Medium	147 (63.1 %)	87 (53.7 %)	22 (40 %)	
High	47 (20.2 %)	59 (36.4 %)	22 (40 %)	
Hypertension^a^	78 (31.2 %)	73 (44.5 %)	22 (39.3 %)	0.021^*^
Body Mass Index category				
<25 kg/m2	62 (24.9 %)	34 (20.1 %)	32 (55.2 %)	<0.000^*^
25-30 kg/m2	141 (56.6 %)	101 (59.8 %)	22 (37.9 %)	
>30 kg/m2	46 (18.5 %)	34 (20.1 %)	4 (6.9 %)	
Waist circumference, cm :				
High (female >88 cm; male >102 cm)	100 (41.3 %)	71 (43.0 %)	30 (52.6 %)	0.299
Obesity^b^	104 (43.5 %)	75 (45.5 %)	30 (52.6 %)	0.462
Family history of CVD^c^	97 (37.6)	61 (39.4)	24 (42.9)	0.754
Diabetes mellitus (DM) type II^d^	10 (4.0)	3 (1.9)	1 (1.8)	0.408
Dyslipidemia^e^	238 (92.6 %)	154 (91.7 %)	56 (94.9 %)	0.715
Health risk behavior:				
Lack of physical activity	107 (42.0)	42 (26.9)	20 (37.0)	0.009^*^
Smoking	42 (16.5)	27 (16.9)	3 (5.4)	0.088
Framingham 10-year CVD risk score category^f^				
Low (<10 %)	153 (66.5 %)	120 (78.9 %)	32 (58.2 %)	0.003^*^
Intermediate (≥10 %, <20 %)	68 (29.6 %)	22 (14.5 %)	20 (36.4 %)	
High (≥20 %)	9 (3.9 %)	10 (6.6 %)	3 (5.5 %)	
Number of inclusion criteria:				
1	51 (19.8 %)	26 (15.4 %)	7 (11.9 %)	0.235
2	68 (26.5 %)	62 (36.7 %)	21 (35.6 %)	
3	77 (30.0 %)	43 (25.4 %)	20 (33.9 %)	
≥4	61 (23.7 %)	38 (22.5 %)	11 (18.6 %)	

^*^statistically different, continuous measurements based on ANOVA test and categorical measurements based on Chi-Square test

^**a**^Hypertension is defined as diastolic blood pressure higher than 90 mmHG or systolic blood pressure higher than 140mmHG or taking antihypertensive drugs

^**b**^Obesity is defined as BMI >30 kg/m^2^ or waist circumference >88 cm for females or >102 cm for males

^**c**^Family history of CVD is defined as a first degree family member who suffered a CVD at any age

^**d**^Diabetes is defined as having a sober blood glucose higher than 6.1 or self-reported diagnosis of diagnosis

^**e**^Dyslipidemia is defined as having increased levels of at least one type of lipids in the blood (total cholesterol ≥ 5 mmol/l, or LDL cholesterol ≥ 2.5 mmol/l; or triglycerides: ≥ 1.7,mmol/l, or HDL cholesterol: ≤ 1.0 mmol/l)

^**f**^EuroSCORE and QRISK2 will be calculated after completion of the intervention, at 12 months

**Table 2:** Baseline characteristics of the study population (demographics, health and work-related factors) in two intervention arms

	Limited Intervention	Extensive Intervention	P value
	*n* = 218	*n* = 275	
Age, mean (sd)	52.1 (6.0)	50.7 (5.4)	<0.006^*^
Organization			0.007
Police	124 (57.1)	138 (50.4)	
Military	60 (27.6)	110 (40.1)	
Hospital	33 (15.2)	26 (9.5)	
Gender:			
Male	154 (77.0 %)	226 (84.6 %)	0.036^*^
Educational level			0.011^*^
Low	33 (17.5 %)	33 (12.6 %)	
Medium	116 (61.4 %)	140 (53.6 %)	
High	40 (21.2 %)	88 (33.7 %)	
Hypertension^a^	73 (35.1 %)	100 (38.2 %)	0.493
Body Mass Index category			
<25 kg/m2	64 (29.9 %)	64 (24.4 %)	0.294
25-30 kg/m2	117 (54.7 %)	147 (56.1 %)	
>30 kg/m2	33 (15.4 %)	51 (19.5 %)	
Waist circumference, cm :			
High (female >88 cm; male >102 cm)	79 (39.7 %)	122 (46.0 %)	0.173
Obesity^b^	81 (40.7 %)	128 (48.9 %)	0.082
Family history of CVD^c^	78 (38.0 %)	104 (39.4 %)	0.767
Diabetes mellitus (DM) type II^d^	8 (3.9 %)	6 (2.3 %)	0.314
Dyslipidemia^e^	197 (91.6 %)	251 (93.3 %)	0.484
Health risk behavior:			
Lack of physical activity	73 (35.6 %)	96 (36.9 %)	0.770
Smoking	23 (11.1 %)	49 (18.6 %)	0.026^*^
Framingham 10-year CVD risk score category^f^			
Low (<10 %)	123 (66.5 %)	182 (72.2 %)	0.430
Intermediate (≥10 %, <20 %)	52 (28.1 %)	58 (23 %)	
High (≥20 %)	10 (5.4 %)	12 (4.8 %)	
Number of inclusion criteria:			
1	43 (20.3 %)	41 (15.0 %)	0.323
2	64 (30.2 %)	87 (31.9 %)	
3	63 (29.7 %)	77 (28.2 %)	
≥4	42 (19.8 %)	68 (24.9 %)	

^*^statistically different, continuous measurements based on ANOVA test, categorical measurements based on Chi-Square test

^**a**^Hypertension is defined as diastolic blood pressure higher than 90 mmHG or systolic blood pressure higher than 140mmHG or taking antihypertensive drugs

^**b**^Obesity is defined as BMI >30 kg/m^2^ or waist circumference >88 cm for females or >102 cm for males

^**c**^Family history of CVD is defined as a first degree family member who suffered a CVD at any age

^**d**^Diabetes is defined as having a sober blood glucose higher than 6.1 or self-reported diagnosis of diagnosis

^**e**^Dyslipidemia is defined as having increased levels of at least one type of lipids in the blood (total cholesterol ≥ 5 mmol/l, or LDL cholesterol ≥ 2.5 mmol/l; or triglycerides: ≥ 1.7,mmol/l, or HDL cholesterol: ≤ 1.0 mmol/l)

^**f**^EuroSCORE and QRISK2 will be calculated after completion of the intervention, at 12 months

Future analyses will be performed according to the Intention-to-treat principle [71]. Repeated measurements with mixed models techniques will be used to compare the primary outcome (general health scores) between the extensive and limited intervention groups, adjusted for potential confounders such as socioeconomic determinants (including employment status, profession, income level, education), and taking into account the cluster randomization (multilevel analysis). A cost analysis will be performed to determine the costs of the extensive health intervention compared to the limited health intervention, including the change in health care consumption.

### Economic evaluation

An economic evaluation will be performed, according to the Dutch guidelines for cost-effectiveness analyses, to evaluate the trade-off between costs and benefits. Analyses will be performed both from the perspective of the organization and from a societal perspective. In this study we will consider direct and induced costs of the intervention. The timeframe of the analyses is 12 months and we will use a model for extrapolation to long-term results. The cost-benefit analysis performed from the organizational perspective will include the differences in employability (in monetary units) and the additional organizational costs for applying the elements from the extensive intervention in addition to the limited intervention. A secondary analysis will be performed in which we will simulate a no-HRA-scenario and calculate the costs and benefits without either one of our interventions as reference. In the cost-effectiveness analyses from a societal perspective, effectiveness will be expressed using QALY’s, whereas costs will be expressed as total healthcare costs (direct plus induced costs) and societal costs. Direct healthcare costs consist of costs of the intervention programs and costs for adapting the intervention for the target groups (including construction and use of the web-portal and MI-training of OPs). Induced healthcare costs consist of healthcare consumption (outpatient visits, diagnostic and therapeutic procedures, medication, and hospital admissions). Societal costs consist of costs of employee absence in order to participate in the intervention, absenteeism, productivity at work, individual costs for undertaking health promotion activities (including contribution fees to a sports centre and time costs), and any additional travel costs and parking expenses.

## Results

### Baseline characteristics of participants

Figure 1 shows the CONSORT diagram [72] of the flow of clusters and participants through the first phases of the trial. In total, 9 clusters (*n* = 217), were assigned to the limited intervention (control), and 9 clusters (*n* = 274) to the extensive intervention (intervention). One cluster dropped out after randomization and before any cardio screenings were done, because the only OP within this cluster had other priorities in work. Three more OPs dropped out before any cardio screenings were done, two because of personal problems and one because she left her job. A total of 652 employees undertook the cardio screening. Based on these cardio screenings, 91.4 % (*n* = 598) were found to have risk factors for CVD, of whom 82.3 % were included (*n* = 493). Reasons for no inclusion were the presence of exclusion criteria or retirement or having to go abroad for work in the near future. For 16 participants the baseline questionnaire was missing, but we did receive blood- or anthropometric-measurements. The number of missings ranged from *n* = 1 (for the variable age) to *n* = 40 (for the variable level of education).

Baseline characteristics of participants in the three organizations are presented in Table 1. The military and police forces included a majority of males, whereas the hospital included mostly females. Among participants of the police force more than 76 % had a BMI higher than 25 kg/m2, and in the military force this percentage was 80.5 %. The percentage in the hospital of 46 % is lower and is similar to the average for the Dutch population.

Table 2 presents the baseline characteristics of the extensive intervention versus control study groups. The average age of the participants was 52, and 81.4 % were male. The randomization was successful in creating study groups with similar characteristics. Only for smoking a difference between the groups was found (*p* = 0.026). The CVD risk based on the Framingham score showed that more than 30 % of our study population is at intermediate or high risk, without differences in distribution between the two groups (*p* = 0.430).

## Discussion

The PerfectFit study aims to establish whether adding MI to a web-based HRA is a key-factor for improving effectiveness in changing unhealthy lifestyles and reducing risk factors for chronic diseases. MI is known to improve BMI, total blood cholesterol, systolic blood pressure, and blood alcohol concentration [73]. By adding MI, this superiority RCT combines the advantages of an online personalized and tailored approach with the advantages of a face-to-face approach, thus aiming to improve participation and sustained effectiveness of workplace health promotion programs [74].

Web-based healthcare (eHealth) is receiving growing attention since it can tailor interventions to target population characteristics (e.g., specific risk factors), thereby facilitating wider access and encouraging self-care, and possibly reducing health-care costs and improving efficacy [75]. However, a proven feature of eHealth is that its effect decreases after the intervention has been completed [15]. Our blended approach of adding MI to a web-based HRA is promising in several ways. First, we expect an increase of lifestyle changes, since previous research showed dose–response relationships between exposure to an intervention (number and duration of exposures) and behavior change outcomes [73, 76]. Second, it may lead to a higher sustainability of these lifestyle changes, since there is evidence that providing face-to-face counselling with a higher number of overall contacts was associated with greater short and long-term effectiveness [76]. MI has a large potential to fill the gap between the intention to change and the actual behavior change, commonly referred to as the intention-behavior gap [77]. This potential was previously demonstrated for lifestyle changes such as physical activity and a healthy diet [78–80]. MI is a unique tool for evoking and strengthening intrinsic motivation for a more sustainable change in lifestyle [13–19].

Our study design is unique for both its blended design of the intervention using a qualified decision aid and the follow-up at 6 and 12 months, which enables us to evaluate not just the initiation of health activities [9] but also the sustainability of lifestyle changes [81]. A second strength is the target population of employees in highly demanding jobs from three types of working organizations, whose workplace productivity and sickness absence might benefit from our intervention. Another strength is that the intervention is implemented within existing health centers and by their own OPs, improving external validity and making the results generalizable to larger companies. For the purpose of this study, a large investment was done for the OPs to master MI, which will remain beneficial to the organizations after completion of the study. Both the OPs from the extensive group and those from the limited group (after completion of the study), are educated to use the MI-technique, and skills they acquire and master during the time of the study and immediately afterwards can be internalized and incorporated in their everyday practice with all employees. From the perspective of the organization and society, these investments may pay off in the long run: an economic evaluation will be performed to analyze whether this truly is the case.

The study has three primary limitations. First, the effects on lifestyle changes may be affected by the healthy volunteer bias, since the study sample consists of employees of organizations who were actively recruited and who volunteered to participate [82]. Although we found differences in characteristics between organizations (Table 1), the potential bias will effect both the intervention and limited group alike, and will not have a large effect on our results (Table 2). Second, an attrition bias may occur at the follow-up measurements since those who achieve lifestyle changes might be more willing to do the final assessment than those who do not [83]. We will try to prevent this by sending personal invitations for final measurements by the OPs and having the OPs stimulate the participant to also fill out the questionnaire. A third issue is that blinding of the OPs, the participants and the investigators would have been preferred but could not be done in this real-life pragmatic trial.

The equal distribution of health factors of our participants between both the intervention and control groups, suggests that we will be able to evaluate the intervention-effects with minimal adjustments. Although there is no statistical difference in BMI between the groups, the percentages of participants that are overweight (BMI > 25 kg/m2) exceeds 70 % in both groups, which is high compared to 47 % within the general population [84]. This is in line with previous research reporting increasingly high percentages of overweight and obesity in the military force [85] and in police officers [86]. However, we are cautiously interpreting overweight in population groups that are doing physically demanding work, since it is well known that muscular type of body composition might lead to higher BMI [87]. Therefore, in our study we also rely on the waist circumference, in order to have an accurate estimation of obesity.

Dyslipidemia, another risk-factor for CVD, exceeds 90 % within both study groups, which could be caused by our strict cut-off value for LDL-cholesterol (>2.5 mmol/l). Nevertheless, since we found that a majority of our participants has more than 1 inclusion criteria and that over 30 % has intermediate or high Framingham risk scores, we can assume that changes in health behaviors can tackle these health and occupational hazards. Actual lifestyle changes will be measured and will also be related to the intention to change at baseline, which will give us a more accurate picture of the participants’ intention-behavior gap.

To keep the workforce vital and productive, there is a growing need for effective and affordable health promotion strategies which can also be easily implemented. A key component of this study is MI, which has several elements that promise to be beneficial for both participants and healthcare providers. MI is a well-described and practical approach that respects individual choices and leads to an increased responsibility for their own health [24, 40]. By adding counselling sessions using MI in the intervention group, participants will be involved in a shared decision making-process related to their lifestyles. We hypothesize that, once participants discover their inner strengths and motivations, their health-behavior gains will be more sustainable and will be maintained over time [15]. Upcoming papers will assess to what extent this hypothesis can be confirmed and whether this blended care approach is an essential strategy for future health promotion programs.

## Abbreviations

Web-based HRA: Web-based Health Risk Assessment
MI: Motivational interviewing
cRCT: cluster Randomized Controlled Trial
CVD: Cardiovascular disease
OP: Occupational health physician
MITI: Motivational interviewing treatment integrity code
SF-36: Short form 36
EuroSCORE: European systematic coronary risk evaluation
WAI: Work ability index
SALSA: Screening of activity limitation and safety awareness
QQ: Quantity and quality
PRODISQ: Productivity of disease questionnaire
WLQ: Work limitations’ questionnaire
EQ-5D: The EuroQoL five dimensions questionnaire
RE-AIM: Reach, effectiveness, adoption, implementation, maintenance
